# Supplementary material for: Correlations between single nucleotide polymorphisms in FABP4 and meat quality and lipid metabolism gene expression in Yanbian yellow cattle
Source: PLoS One. 2020 Jun 24;15(6):e0234328. doi: 10.1371/journal.pone.0234328 (PMC7314053; doi:10.1371/journal.pone.0234328)
Supplement: S2 Fig — (DOCX) [file pone.0234328.s002.docx]

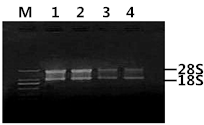


**S2 Fig. Agarose gel electrophoresis of RNA.** M: DL2000 DNA marker (Takara Biomedical Technology (Beijing) Co., Ltd). 1-4: Genomic RNA samples from the blood of Yanbian yellow cattle.
