# Supplementary material for: Correlations between single nucleotide polymorphisms in FABP4 and meat quality and lipid metabolism gene expression in Yanbian yellow cattle
Source: PLoS One. 2020 Jun 24;15(6):e0234328. doi: 10.1371/journal.pone.0234328 (PMC7314053; doi:10.1371/journal.pone.0234328)
Supplement: S3 Fig — (DOCX) [file pone.0234328.s003.docx]

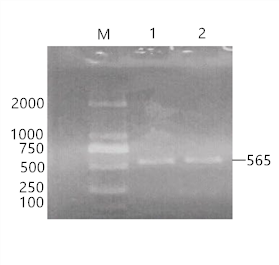


**S3 Fig. Un-cropped images of electrophoretic map analyses shown in Fig. 2.** M: DL2000 DNA marker (Takara Biomedical Technology (Beijing) Co., Ltd). 1 and 2: PCR products of *FABP4* gene. This image was obtained by the Gel Imaging Analyzer of Shanghai PeiQing Technology co.,Ltd.
