# Supplementary material for: Correlations between single nucleotide polymorphisms in FABP4 and meat quality and lipid metabolism gene expression in Yanbian yellow cattle
Source: PLoS One. 2020 Jun 24;15(6):e0234328. doi: 10.1371/journal.pone.0234328 (PMC7314053; doi:10.1371/journal.pone.0234328)
Supplement: S4 Fig — (DOCX) [file pone.0234328.s004.docx]

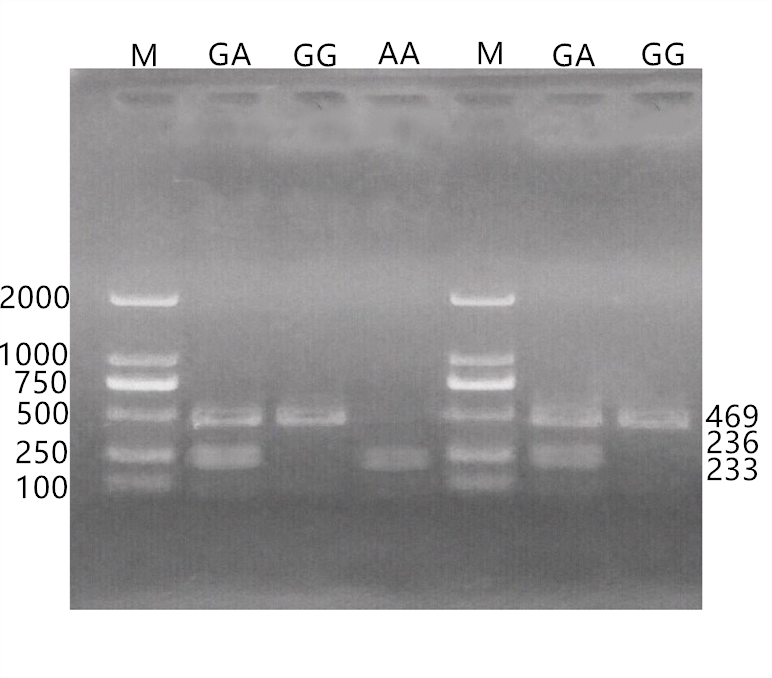


**S4 Fig. Un-cropped images of electrophoretic map analyses shown in Fig. 9.** M: DL2000 DNA marker (Takara Biomedical Technology (Beijing) Co., Ltd). GA, GG, and AA represent the three genotypes of *FABP4* detected by sequencing. This image was obtained by the Gel Imaging Analyzer of Shanghai PeiQing Technology co.,Ltd.
