# Supplementary material for: Correlations between single nucleotide polymorphisms in FABP4 and meat quality and lipid metabolism gene expression in Yanbian yellow cattle
Source: PLoS One. 2020 Jun 24;15(6):e0234328. doi: 10.1371/journal.pone.0234328 (PMC7314053; doi:10.1371/journal.pone.0234328)
Supplement: S6 Fig — (DOCX) [file pone.0234328.s006.docx]

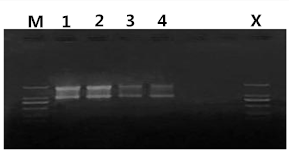


**S6 Fig. Un-cropped images of electrophoretic map analyses shown in S2 Fig.** M: DL2000 DNA marker (Baori Medical Biotechnology Co., Ltd., Dalian). 1-4: RNA sample of longus dorsal muscle of Yanbian yellow cattle. This image was obtained by the Gel Imaging Analyzer of Shanghai PeiQing Technology co.,Ltd.
