# Supplementary material for: Correlations between single nucleotide polymorphisms in FABP4 and meat quality and lipid metabolism gene expression in Yanbian yellow cattle
Source: PLoS One. 2020 Jun 24;15(6):e0234328. doi: 10.1371/journal.pone.0234328 (PMC7314053; doi:10.1371/journal.pone.0234328)
Supplement: S1 File — (DOC) [file pone.0234328.s007.doc]

**S1 File. Procedure for the Blood Genomic DNA Extraction Kit.** The instructions of Blood genomic DNA Extraction Kit (0.1-1ml) (DP318) of Tiangen biochemical technology (Beijing) Co., Ltd.

**Steps:**

**Before use, add anhydrous ethanol to buffer GD and bleach PW. Please refer to the label on the bottle.**

1. Treatment of blood materials (**This product is suitable for treatment of 100 μl-1 ml blood samples with anticoagulant added**) :

A. When the volume of blood sample is less than 200 μl, buffer solution GS can be added to make up the volume to 200 μl, and then the next experiment can be conducted (**If the volume of blood sample is 200 μl, the next experiment can be conducted directly without adding GS**).

B. When blood sample volume of more than 200 μl, need a cell lysis liquid CL processing, specific steps are as follows:

Add 1 to 2.5 times the volume in the sample cell lysis of liquid CL, reverse blending, 10000 rpm centrifugal 1 min, absorb supernatant, leaving the nucleus precipitation (If cracking is not complete, can you repeat the steps again), is added into centrifugal nucleus precipitation collected 200 μl buffer GS, oscillation to thorough blending.

C. If the treated blood samples are the anticoagulant blood of birds, amphibians or lower organisms, and the red blood cells are nucleated fine cells, the treatment capacity is 5-20 μl, and buffer solution GS can be added to supplement 200 μl and the following steps of lysis can be performed.

**Note: If the RNA needs to be removed, add 4 μl of RNase A (100 mg/ml) solution (customer-provided, Directory NO.: RT405-12), oscillate for 15 sec, and leave at room temperature for 5 min.**

2. Add 20 μl of Proteinase K solution and mix well.

3. Add 200 μl of buffer solution GB, mix thoroughly upside down, and place it at 56 °C for 10 min. Mix upside down several times, and the solution will be strained and bright **(If the solution is not completely clear, please extend the cracking time until the solution is clear).**

**Note: white precipitate may occur when buffer GB is added, which generally disappears when placed at 37 °C and will not affect the subsequent test. If the solution is not clear, it indicates that the cell lysis is not complete, which may lead to less extracted DNA and the extracted DNA is not pure.When the blood volume is less than 200 μl and no erythrocyte lysis treatment is adopted, or the sample storage condition is not good, the color may be dark brown after the water bath, note that there is no clumps and other precipitation in the solution.**

4. Add 200 μl of anhydrous ethanol, mix thoroughly upside down, and a flocculent precipitate may appear.

5. The solution and flocculent precipitation obtained in the previous step were added to an adsorption column CB3 (the adsorption column CB3 was put into the collection tube) and centrifuged at 12,000 rpm for 30 sec. The waste liquid in the collection tube was dumped and the adsorption column CB3 was put into the collection tube.

6. Add 500 μl buffer GD to the adsorption column CB3 (**Please check whether anhydroethanol has been added before using**), centrifuge 30 sec at 12,000 rpm, discard the waste liquid in the collection tube, and put the adsorption column CB3 into the collection tube.

7. Add 600 μl rinsing solution PW to the adsorption column CB3 (**Please check whether anhydric ethanol has been added before use**), centrifuge it at 12,000 rpm for 30 sec, discard the waste liquid in the collection tube, and put the adsorption column CB3 into the collection tube.

8. Repeat step 7.

9. Centrifuge at 12,000 rpm for 2 min, and discard the waste liquid. The adsorption column CB3 was placed at room temperature for a few minutes to dry the residual bleach solution in the adsorption material thoroughly.

**Note: the purpose of this step is to remove the residual rinsing solution in the adsorption column. The ethanol residue in the rinsing solution will affect the subsequent enzyme reactions (enzyme digestion, PCR, etc.).**

1. Transfer the adsorption column CB3 into a 1.5 ml centrifuge tube, suspend drops of 50-200 μl of elution buffer TB to the middle position of the adsorption film, place them at room temperature for 2-5 min, centrifuge the solution at 12,000 rpm for 2 min, and collect the solution into the centrifuge tube.

**Note: the elution buffer liquid volume should not be less than 50 μl. In order to increase the yield of genomic DNA, the solution obtained by centrifugation can be added to the adsorption column CB3, placed at room temperature for 2 min, and centrifuged at 12,000 rpm for 2 min.The pH of the eluent has a great influence on the eluent efficiency. If the eluent is made with ddH_2_O, the pH value should be within the range of 7.0-8.5. If the pH value is lower than 7.0, the eluent efficiency will be reduced. DNA products should be stored at -20 °C to prevent DNA degradation.**
