# Supplementary material for: Correlations between single nucleotide polymorphisms in FABP4 and meat quality and lipid metabolism gene expression in Yanbian yellow cattle
Source: PLoS One. 2020 Jun 24;15(6):e0234328. doi: 10.1371/journal.pone.0234328 (PMC7314053; doi:10.1371/journal.pone.0234328)
Supplement: Attachment — Submitted filename: Response to Reviewers.doc [file pone.0234328.s009.doc]

1. Please make sure that the paper adheres to English grammar throughout the text.

Response: We have asked Editage to get a native English speaker to revise the entire manuscript.

2. To enhance the reproducibility of your results, we recommend that if applicable you deposit your laboratory protocols in protocols.io, where a protocol can be assigned its own identifier (DOI) such that it can be cited independently in the future.

Response: We have updated the protocol of our experiment according to the newly revised manuscript. The DOI address is [dx.doi.org/10.17504/protocols.io.bgnjjvcn](https://dx.doi.org/10.17504/protocols.io.bgnjjvcn).

3. There are several errors of the English grammar which to be eliminated prior to acceptance for publication. I urge the authors to contact a native English speaker.

Response: We have asked Editage to get a native English speaker to revise the entire manuscript.

4. While revising your submission, please upload your figure files to the Preflight Analysis and Conversion Engine (PACE) digital diagnostic tool.

Response: As requested by your magazine, we have uploaded the graphics file to PACE.
